# Calpain-2 Facilitates Autophagic/Lysosomal Defects and Apoptosis in ARPE-19 Cells and Rats Induced by Exosomes from RPE Cells under NaIO_3_ Stimulation

**DOI:** 10.1155/2023/3310621

**Published:** 2023-01-17

**Authors:** Shuaishuai Zhang, Yingzhe Qiu, Yuan Feng, Yi Zhang, Yanan Li, Boxin Wang, Heliang Wei, Xilong Chen, Lixia Shen, Wei Li, Liqing Zheng, Yuanyuan Zhang

**Affiliations:** ^1^Shenyang Pharmaceutical University, Shenyang 110016, China; ^2^Department of Pharmacy, Hebei North University, Hebei Key Laboratory of Neuropharmacology, Zhangjiakou 075132, China

## Abstract

Although accumulated evidence supports the notion that calpain contributes to eye disease, the mechanisms by which calpain promotes RPE injury are not defined. The present study is aimed at investigating whether the effect of NaIO_3_-exos (exosomes derived from RPE cells under NaIO_3_ stimulation) on the dysfunction of the autophagy-lysosomal pathway (ALP) and apoptosis is based on its regulation of calpain activation in ARPE-19 cells and rats. The results showed that calpain-2 activation, ALP dysfunction, and apoptosis were induced by NaIO_3_-exos in ARPE-19 cells. NaIO_3_-exo significantly increased autophagic substrates by activating lysosomal dysfunction. ALP dysfunction and apoptosis in vitro could be eliminated by knocking down calpain-2 (si-C2) or the inhibitor calpain-2-IN-1. Further studies indicated that NaIO_3_-exo enhanced calpain-2 expression, ALP dysfunction, apoptosis, and retinal damage in rats. In summary, these results demonstrate for the first time that calpain-2 is one of the key players in the NaIO_3_-exo-mediated ALP dysfunction, apoptosis, and retinal damage and identify calpain-2 as a promising target for therapies aimed at age-related macular degeneration (AMD).

## 1. Introduction

Age-related macular degeneration (AMD) is a progressive neurodegenerative disease with a high rate of blindness, which affects vision in older individuals worldwide [1]. AMD is subdivided into two forms: wet AMD (about 5%) and dry AMD (more than 90%). The degeneration of the retinal pigment epithelial (RPE), which results in the secondary death of photoreceptors and drusen, is the primary feature observed in dry AMD. However, wet AMD is characterized by choroidal neovascularization (CNV) [2]. RPE loss may also cause loss of the choriocapillaris. Therefore, RPE plays a key role in AMD [3].

RPE cells can secrete exosomes in a paracrine manner to interfere with neighboring cells. Furthermore, the pathological process of eye diseases could be influenced by exosomes delivered via intranasal, intraocular, intravenous, and other mechanisms [4]. The role of RPE-derived exosomes in a potentially harmful oxidative response has been well established under photooxidative blue-light stimulation conditions [5]. Exosomes secreted by rotenone could increase cell apoptosis, oxidative injury, and inflammation in ARPE-19 cells [6]. RPE cell necrosis followed by photoreceptor cell apoptosis and choriocapillaris was evoked in NaIO_3_-treated mice [7]. However, the role of exosomes induced by NaIO_3_ (NaIO_3_-exo) in RPE insult is unknown.

The pathological changes including apoptosis, autophagy, and oxidative stress occur during the AMD process. Autophagy is an evolutionarily conserved and lysosome-dependent protein degradation process. AMD is usually associated with autophagy-lysosomal pathway (ALP) dysregulation [8]. We have previously found that Earle's balanced salt solution (EBSS) induced autophagy in ARPE-19 cells, whereas the detailed roles of the dynamic changes in the autophagy-lysosomal function remain unclear [9]. Calpains can be activated by calcium influx and play critical roles in neurodegenerative disease [10–12]. Calpain-2 is usually identified as the major harmful form of calpain in AMD. Activated calpain-2 has been shown to break down autophagy-related proteins and mitochondrial membrane proteins, which may participate in EBSS-induced RPE death [13]. It has been shown that lysosomal and autophagy defects caused by calpain-induced lysosomal membrane permeabilization (LMP) are closely associated with the loss of the polycystic kidney disease gene 1 (PKD1) in kidney disease [14]. Evidence has shown that calpain-2 can modulate autophagy by cleaving the autophagy-related proteins ATG3 (autophagy related 3) and ATG7 (autophagy related 7) in ischemia–reperfusion injury [15]. Calpain-2 also plays a pivotal role in the tumor necrosis factor- (TNF-) *α*-induced autophagy in mouse hippocampal neurons [16]. Moreover, the tunicamycin-mediated calpain-2-dependent endoplasmic reticulum stress pathway leads to hepatic stellate cell apoptosis [17]. In addition, it has been reported that calpain-2 is involved in neuronal apoptosis induced by polybrominated diphenyl ether-153 [18]. However, the role of calpain-2 in regulating ALP function and apoptosis in NaIO_3_-exo-induced RPE insult is so far unknown.

In the present study, we investigated the role of calpain-2 in regulating ALP functions and apoptosis in NaIO_3_-exo-induced RPE insult in ARPE-19 cells and rats. First, the goal was to explore the effects of NaIO_3_-exo on the calcium flux, autophagic flux defects, lysosome dysfunction, and apoptosis in ARPE-19 cells. Chloroquine (CQ) and 3-methyladenine (3-MA) were used to confirm whether the increase in autophagic substrates was due to lysosomal dysfunction. Knocking down of the calpain-2 or inhibitor calpain-2-IN-1 was used to study the possible role of calpain-2 on the dynamic changes of the ALP function and apoptosis in ARPE-19 cells. Finally, NaIO_3_-exo-induced calpain-2 activation, ALP dysfunction, apoptosis, and retinal damage were evaluated in rats. Our data show that NaIO_3_-exos induce autophagic/lysosomal defects and apoptosis through calpain-2 in ARPE-19 cells and rats.

## 2. Materials and Methods

### 2.1. Cell Culture and Treatment

ARPE-19 cells, purchased from Shanghai GuanDao Biotech Co. Ltd., were maintained in Dulbecco's Modified Eagle's Medium (DMEM)/F-12 (Hyclone, Logan, UT, USA) containing 10% free exosome FBS (Gibco, Grand Island, NY, USA). Cells that had grown to 80% confluence were used in the experiments. ARPE-19 cells were subcultured in 6-well plates at a seeding density of 2 × 10^5^ cells/well for 24 h. Then, the cells were exposed to NaIO_3_-exos (40 *μ*g/ml) for 6, 12, or 24 h for our experiments. The cultures were exposed to con-exos (40 *μ*g/ml) or normal medium for 24 h and used as negative controls.

### 2.2. Exosome Isolation and Characterization

The NaIO_3_-induced ARPE-19 cells and untreated ARPE-19 cells were centrifuged at 4°C and 300 g for 10 minutes. Cell culture supernatants were collected and centrifuged at 4°C sequentially for 10 min at 2000 g, 30 min at 10 000 g, and 70 min at 100 000 g. The suspension was filtered through a 0.22 *μ*m membrane and centrifuged again for 70 min at 100 000 g (Optima XPN-100 Ultracentrifuge, Beckman, USA). The exosome secreted from untreated ARPE-19 cells was named the con-exo group, while the exosome secreted from the NaIO_3_-induced ARPE-19 cells was named the NaIO_3_-exo group. The exosome concentration was measured using a BCA protein assay kit (Beyotime Biotech). The final exosome concentration was 1.48 mg·ml^−1^. The morphology of the exosomes was observed and photographed using a transmission electron microscope (H-7650 TEM, Hitachi, Japan). Particle size distribution of exosomes was analyzed by Nanoparticle Tracking Analysis (NTA). Potential surface markers were examined by western blotting and flow cytometry assay.

### 2.3. Flow Cytometry

The results from the flow cytometric analysis of exosomes labeled with TSG101 (ab125011) or CD63 (ab271286) were compared to a rabbit monoclonal IgG (ab172730) isotype control. The cytosolic-free calcium was detected using Fluo-3 AM. The number of dead cells was determined by FITC-AnnexinV (AV)/propidium iodide (PI) double staining (Absin, Shanghai, China, ABS50001A).

### 2.4. Immunofluorescence

Cells were fixed with 4% paraformaldehyde (PFA). After extensive washing, the cells were incubated overnight at 4°C with primary antibodies LAMP1 (1 : 500; Abcam, ab24170), LC3 (1 : 1000; Abcam, ab63817), and DAPI (1 : 200; Beyotime Biotech) in PBS. Next, cells were washed and incubated with a fluorochrome-coupled secondary antibody (1 : 200; Beyotime Biotech, Alexa Fluor 488, Alexa Fluor 594). The images were taken using a confocal microscope (Olympus; Tokyo, Japan). Images were analyzed with the Image-Pro Plus 6.0 software (Media Cybernetics, Silver Spring, MD).

### 2.5. Cell Transfection

To silence calpain-2, ARPE-19 cells were transfected with *calpain-2* siRNA or nonspecific siRNA using Lipofectamine 2000 for 12 h. After changing the medium, the cells were incubated for another 48 h. The control sequence was 5′-UUCUCCGAACGUGUCACGUTT-3′. The specific siRNA against *calpain-2* was 5′-GAAGUGGAAACUCACCAAATT-3′. Transfection efficiency was evaluated by western blotting (GenePharma; Shanghai, China).

### 2.6. Western Blotting

The protocol of the western blot analysis is described in detail elsewhere [9]. The primary antibodies were as follows: calpain-1 (1 : 1000; Cambridge, MA, USA, ab108400), calpain-2 (1 : 1000; Abcam, ab126600), LAMP1 (1 : 1000; Abcam, ab24170), CTSB (1 : 1000; Millipore, 06480-1), LC3 (1 : 1000; Abcam, ab63817), Beclin-1 (1 : 1000; CST, Technology, Boston, MA, USA, #3495), P62 (1 : 1000; Abcam, ab109012), ATG5 (1 : 500; CST, #12994), TSG101 (1 : 1000; Abcam, ab125011), CD63 (1 : 1000; Abcam, ab271286), V-ATPase (1 : 500; Abcam, ab105937), VAMP7 (1 : 1000; CST, #13920), and *β*-actin (1 : 500; Proteintech, Chicago, USA, 66009).

### 2.7. Animals and NaIO_3_-Exo-Induced AMD Rat Model

6–8 week-old male Sprague-Dawley (SD) rats for the AMD model were supplied by the Experimental Animal Centre of the Shenyang Pharmaceutical University. All experiments were carried out according to the Regulations of the Experimental Animal Administration issued by the State Committee of Science and Technology of China. Approval for this study was obtained from the Institutional Ethics Review Board of Shenyang Pharmaceutical University.

The rats were randomly divided into three groups: control group, con-exo group, and NaIO_3_-exo group. Rats were anesthetized by intraperitoneal injection of 1% pentobarbital sodium salt (30 mg/kg), supplemented with topical application of proparacaine hydrochloride (0.5%; provided by Zhongshan University). 5 *μ*L con-exo or NaIO_3-_exo was injected into the subretinal cavity of the right eye of each animal in the con-exo and the NaIO_3_-exo groups. The eyes of the controls were injected with saline solution as previously reported [19]. Ofloxacin eye ointment was applied to the rats after each injection to prevent infection, and the animals were examined with a slit lamp.

### 2.8. Exosome Tracking

Before injection, exosomes were incubated with molecular probes (Vybrant™ DiO) at a final concentration of 1 *μ*M at 37°C for 30 min. The labeled exosomes were isolated by Exoquick-TC (System Biosciences) and centrifuged at 14 000 g for 30 minutes. The exosomes were washed with PBS and resuspended in PBS on ice. Exosomes were recorded on days 0, 7, and 14 after injection. All retina images were obtained using a confocal microscope (Olympus, Tokyo, Japan).

### 2.9. Hematoxylin and Eosin (HE) Staining

The rats were euthanized two weeks after injection. Their eyes were perfused and stored in Bouin's solution (*n* = 4/group). The eyes were embedded in paraffin after generating 4 *μ*m-thick serial sections. The eye sections were processed according to the standard procedure and then stained with H&E staining. The images were acquired using a light microscope (Olympus BX40, Tokyo, Japan).

### 2.10. TUNEL Assay

Cell apoptosis rate in ARPE-19 cells and rat retinal tissues was visualized by using the terminal deoxynucleotidyl transferase dUTP nick end labeling (TUNEL) assay with an apoptosis detection kit (Roche, Mannheim, Germany). The TUNEL assay was performed according to the manufacturer's protocol.

### 2.11. Statistics

Statistical analysis was performed using SPSS 21.0 (SPSS Inc., Chicago, IL) and GraphPad Prism 6.0 (GraphPad Software Inc). Results are reported as the mean ± standard error of the mean (SEM). One-way ANOVA and Dunnett's posttest were used to determine the statistical significance. The values were considered significant when *P* was <0 05.

## 3. Results

### 3.1. The Characterization of Exosomes Secreted by ARPE-19 Cells or NaIO_3_-Stimulated ARPE-19 Cells

**Figure 1:**
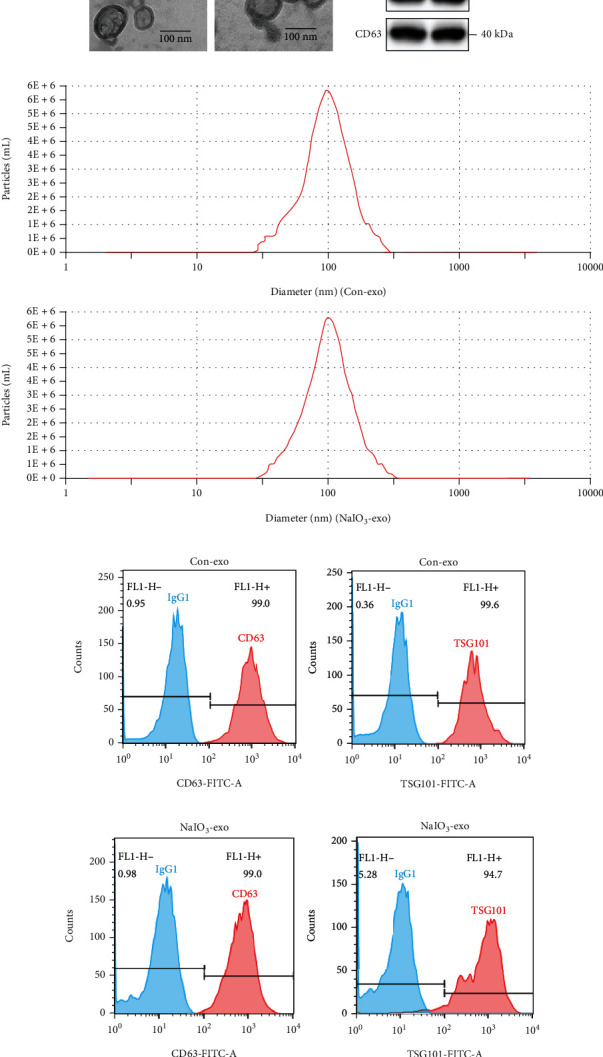
The characterization of exosomes secreted by ARPE-19 cells or NaIO_3_-stimulated ARPE-19 cells. (a) Exosome morphology detected by TEM. (b) The expression levels of the specific exosomal biomarker TSG101 and CD63 proteins were examined by western blotting. (c) Diameter distribution of the particles detected by NTA (nm). (d) Flow cytometry results indicated that the exosomes in the control group were CD63 (99.0%) and TSG101 (99.6%) positive. (e) Flow cytometry results indicated that the exosomes were CD63 (99.0%) and TSG101 (94.7%) positive in the NaIO_3_ group.

The morphology of exosomes secreted by untreated ARPE-19 cells or NaIO_3_-stimulated ARPE-19 cells was examined using TEM. The TEM images showed that the exosomes were cup-shaped with diameters between 50 and 150 nm (Figure 1(a)). Western blotting demonstrated that the exosomal protein markers of TSG101 and CD63 were highly expressed in isolated particles (Figure 1(b)). Consistent with the findings from TEM, NTA results indicated that the particle diameter ranged from 50 to 150 nm (Figure 1(c)). Additionally, flow cytometric analysis revealed that the positivity rates of CD63 and TSG101 in the control group are 99.0% and 99.6%, respectively (Figure 1(d)). The positivity rates of CD63 and TSG101 in the NaIO_3_ group are 99.0% and 94.7%, respectively (Figure 1(e)).

### 3.2. Exosome (NaIO_3_) Induced Calcium Overload, Autophagy, and Apoptosis in ARPE-19 Cells

**Figure 2:**
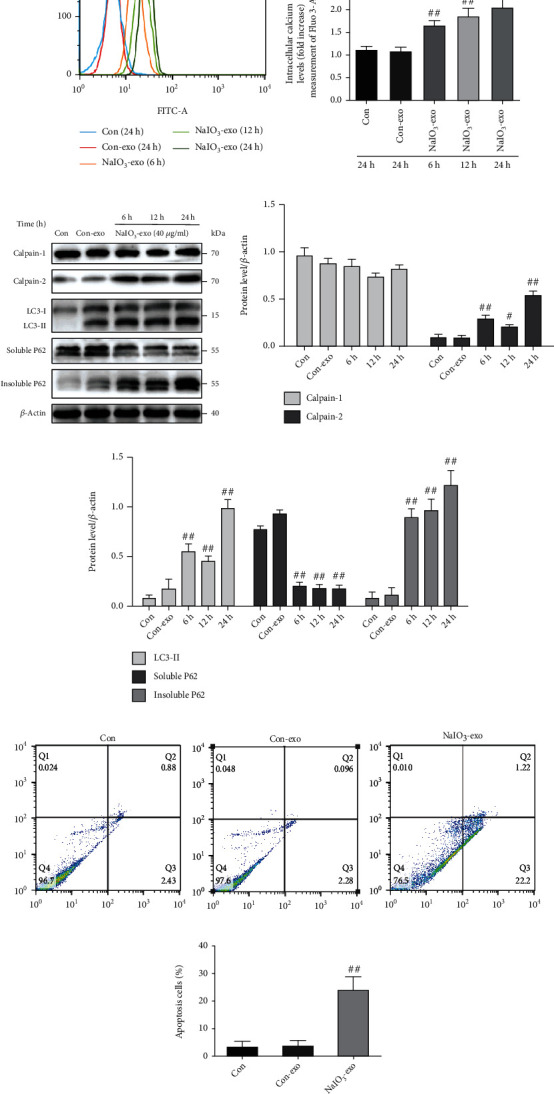
The effects of exosomes (NaIO_3_) on calcium fluxes, calpain activation, autophagic flux defects, and apoptosis in ARPE-19 cells. (a, b) The cytosolic-free calcium was detected using a flow cytometer. (c–e) Western blot analysis was performed to detect the expression levels of calpain-1, calpain-2, and autophagy-related proteins in ARPE-19 cells. (f, g) The apoptosis rate of ARPE-19 cells was evaluated by flow cytometry. ^#^*P* < 0.05, ^##^*P* < 0.01 vs. Con (control group).

First, our data showed no significant difference in calcium flux, autophagic flux, and lysosome function between the control group and the con-exo group at 0 h, 6 h, and 24 h. Therefore, a time period of 24 h was selected for our experiments (Figure 1 in the Data Supplement). To explore the role of NaIO_3_-exo on calcium flux in the ARPE-19 cells, the extracted con-exos (40 *μ*g/ml) were cocultured with ARPE-19 cells for 24 h, and the extracted NaIO_3_-exos (40 *μ*g/ml) were cocultured with ARPE-19 cells for 6 h, 12 h, and 24 h. The data showed that NaIO_3_-exo significantly increased cytosolic-free calcium compared with the control group, which suggests that NaIO_3_-exo triggered calcium overload in the ARPE-19 cells (Figures 2(a) and 2(b)). P62 levels in both Triton X-100-soluble and Triton X-100-insoluble fractions, autophagic marker LC3, and calpain-1/2 were detected by western blotting. The results showed that NaIO_3_-exo markedly increased the expression of calpain-2, the autophagic marker LC3II, and the insoluble P62 in ARPE-19 cells in the time frame of 6 to 24 h, which is consistent with the effects of NaIO_3_ (Figures 2(c)–2(e), Figure 2C-E in the Data Supplement). Our previous study had demonstrated that NaIO_3_ increased the cell apoptosis rate at 24 h in ARPE-19 cells (Figure 2F, G in the Data Supplement). Consistently, NaIO_3_-exo significantly promoted apoptosis in ARPE-19 cells compared with the control group (Figures 2(f) and 2(g)). Our data suggest that NaIO_3_-exo induced calpain activation, autophagy, and cell apoptosis in ARPE-19 cells.

### 3.3. Exosome (NaIO_3_) Induced Lysosome Dysfunction in ARPE-19 Cells

**Figure 3:**
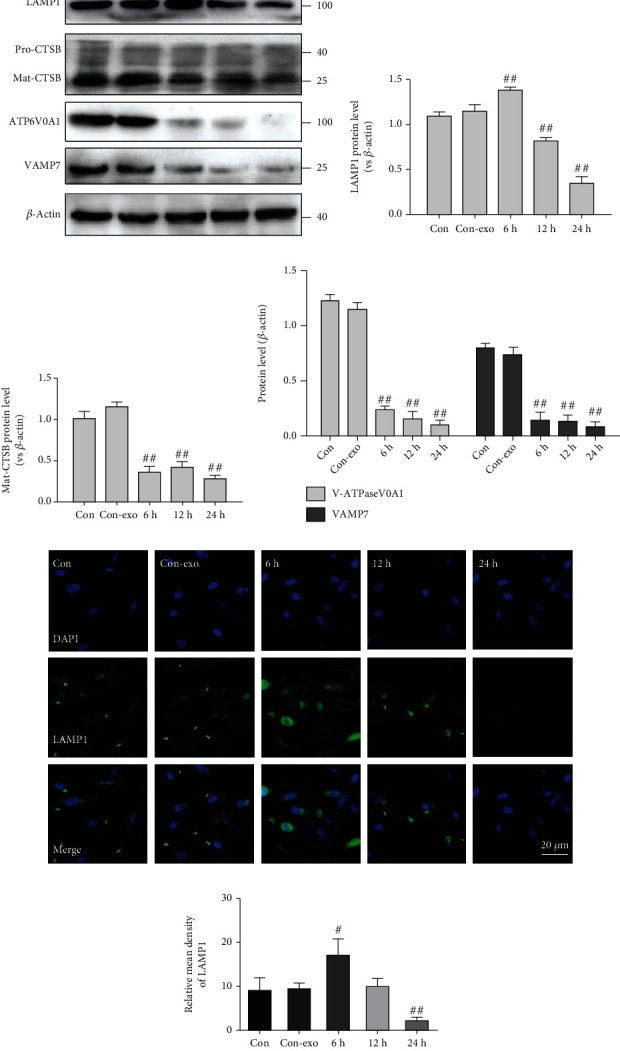
Exosome (NaIO_3_) induced lysosome dysfunction in ARPE-19 cells. (a–d) The levels of LAMP1, CTSB, ATP6V0A1, and VAMP7 were detected by western blotting. (e, f) Confocal microscopy images of ARPE-19 cells labeled with anti-LAMP1 (green) and nuclear-stained labeled with DAPI (blue). ^#^*P* < 0.05, ^##^*P* < 0.01 vs. Con (control group).

The lysosome is an essential organelle for the digestion of autophagic substrates [20]. To further explore whether NaIO_3_-exo induces autophagosome accumulation by increasing the formation or decreasing the degradation of autophagosomes, we examined changes in the expression of the lysosomal markers LAMP1 and CTSB. The protein expression of Mat-CTSB decreased from 6 h until 24 h, and the expression of LAMP1 significantly increased at 6 h and then gradually decreased compared with the control group, indicating that lysosomes ruptured at the late stage of the NaIO_3_-exo action. V-ATPase, a proton pump that is responsible for establishing and maintaining the acidic environment of lysosomes, was also examined. The findings suggest that the expression of ATP6V0A1 significantly decreased compared with the control group. Moreover, the expression of VAMP7, a member of the SNARE complex, responsible for the fusion of lysosomes with autophagosomes, significantly decreased (Figures 3(a)–3(d)) [21, 22]. We also assessed lysosomal function by using confocal microscopy to examine LAMP1. Consistent with the western blotting results, immunofluorescence analysis of LAMP1 showed that the fluorescence signal initially increased and then decreased again (Figures 3(e) and 3(f)). These findings reveal that NaIO_3_-exo induced marked lysosomal dysfunction in ARPE-19 cells.

### 3.4. The Effect of Exosomes (NaIO_3_) on the Defects in ALP due to Lysosome Dysfunction

**Figure 4:**
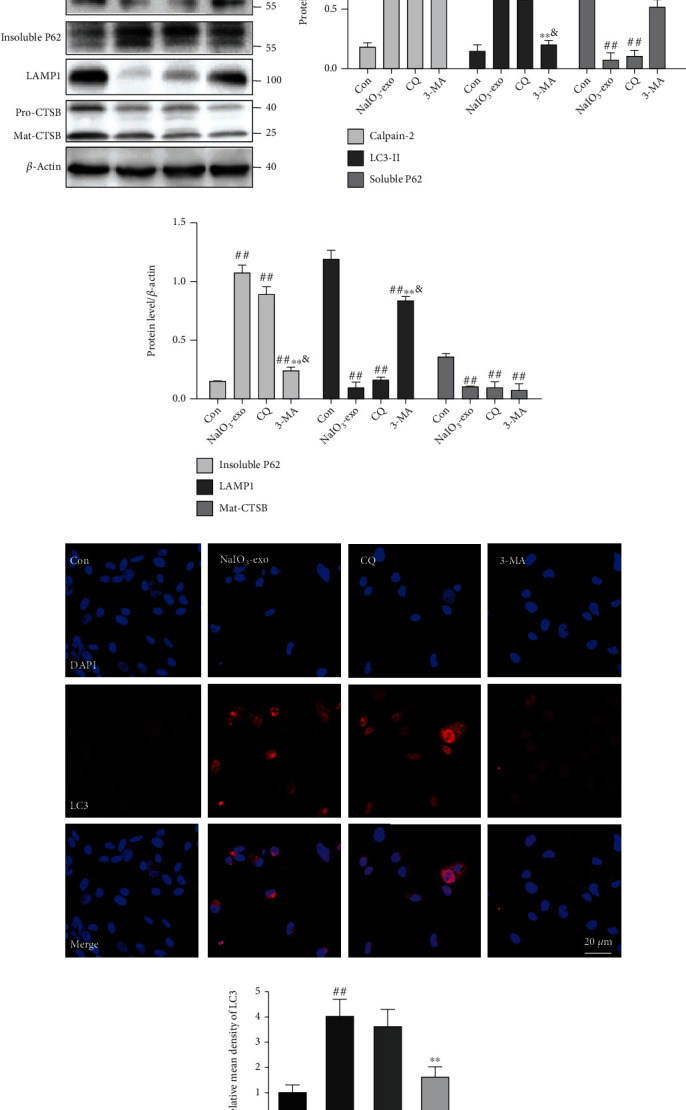
The effects of CQ or 3-MA on the ALP dysfunction and calpain-2 in ARPE-19 cells. (a–c) Calpain-2 and ALP-related proteins were examined by western blotting. (d, e) Representative immunofluorescence images and quantitative analysis of autophagosomes (LC3-positive cells shown in red). ^#^*P* < 0.05, ^##^*P* < 0.01 vs. Con (control group). ^∗^*P* < 0.05, ^∗∗^*P* < 0.01 vs. NaIO_3_-exo group. ^&^*P* < 0.05 vs. CQ group.

To determine whether the effect of NaIO_3_-exo on ARPE-19 cells was related to autophagy-lysosomal pathway (ALP) dysfunction, we investigated the ALP-related proteins in the presence of two different autophagy inhibitors, 3-methyladenine (3-MA, 1 mM) or chloroquine (CQ, 40 *μ*M). The data showed that NaIO_3_-exo caused increased LC3II and insoluble P62 levels as compared with the control group, while downregulating LAMP1, Mat-CTSB, and soluble P62 levels. These levels were not altered in the presence of CQ, suggesting that the autophagic flux was markedly impaired. However, 3-MA markedly decreased the LC3II and insoluble P62 levels, and the expression levels of LAMP1 and Mat-CTSB were increased. No change was observed in the calpain-2 level in the presence of NaIO_3_-exo, CQ, or 3-MA (Figures 4(a)–4(c)). Similarly, confocal microscopy analysis showed that NaIO_3_-exo dramatically increased the expression of LC3II, but 3-MA slightly decreased the LC3II level (Figures 4(d) and 4(e)). These data confirmed that the increase in autophagic substrates was due to lysosomal dysfunction.

### 3.5. Calpain-2 Involvement in the Promotion of ALP Function and Apoptosis in ARPE-19 Cells

**Figure 5:**
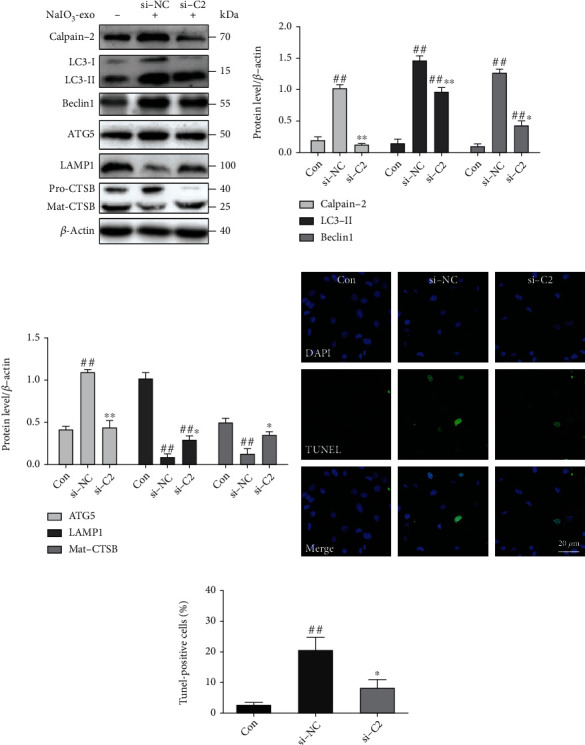
Knockdown of the calpain-2 enhanced ALP function and attenuated apoptosis in ARPE-19 cells. (a–c) Western blotting showed the expression of proteins related to ALP and calpain-2. (d, e) Knockdown of calpain-2 reduced apoptotic cells (green). ^##^*P* < 0.01 vs. Con (control). ^∗^*P* < 0.05, ^∗∗^*P* < 0.01 vs. si-NC group.

**Figure 6:**
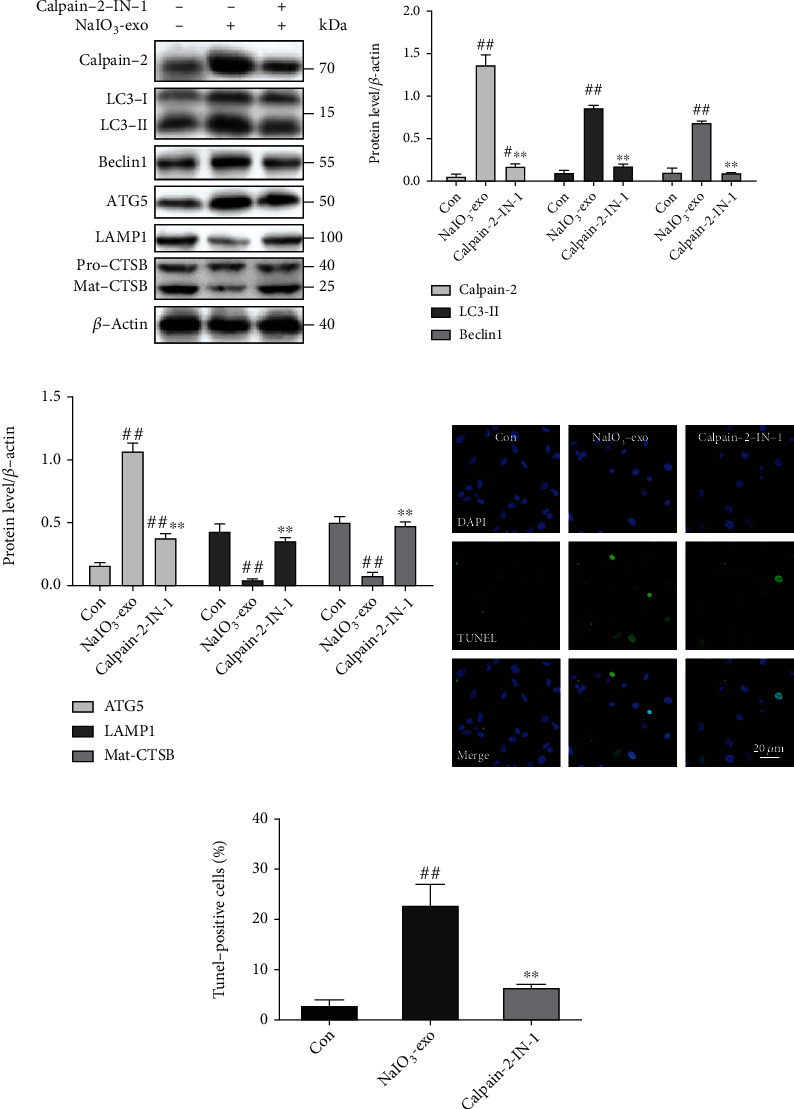
Inhibition of calpain-2 by calpain-2-IN-1 attenuated lysosomal membrane permeabilization, autophagosome formation, and apoptosis in ARPE-19 cells. (a–c) The expression of calpain-2 and ALP-related proteins was examined by western blotting. (d, e) The apoptotic cells were measured by TUNEL. ^#^*P* < 0.05, ^##^*P* < 0.01 vs. Con (control). ^∗∗^*P* < 0.01 vs. NaIO_3_-exo group.

The knocking down of calpain-2 (si-C2) or the inhibitor calpain-2-IN-1 was used to further explore the possible role of calpain-2 on the dynamic changes of the ALP function and apoptosis in ARPE-19 cells. It has been demonstrated that calpain-2 cleaves ATG proteins [15]. Therefore, we also examined the expression of Beclin1 and ATG5. The data showed that the protein levels of calpain-2, Beclin1, ATG5, and LC3II were significantly upregulated in the si-NC or NaIO_3_-exo groups, relative to the control group. Inhibition of calpain-2 by genetic knockdown with si-C2 or the pharmacological inhibitor calpain-2-IN-1 markedly decreased the expression of calpain-2, Beclin1, ATG5, and LC3II, suggesting that calpain-2 activation can promote autophagosome accumulation. We also examined lysosome function under calpain-2 inhibition conditions. Inhibition of calpain-2 by either si-C2 or calpain-2-IN-1 increased the expression of LAMP1 and Mat-CTSB (Figures 5(a)–5(c) and 6(a)–6(c)). In addition, we further examined the role of calpain-2 on ARPE-19 cell apoptosis. The TUNEL assay revealed that the apoptosis rate significantly increased in the si-NC or NaIO_3_-exo group but was ameliorated by the si-C2 or calpain-2-IN-1 treatment (Figures 5(d), 5(e), 6(d), and 6(e)). These results provide evidence that NaIO_3_-exo–activated calpain-2 participates in the ALP function and ARPE-19 cell apoptosis.

### 3.6. Exosome (NaIO_3_) Induced Calpain-2 Activation, ALP Dysfunction, Apoptosis, and Retinal Damage in Rats

**Figure 7:**
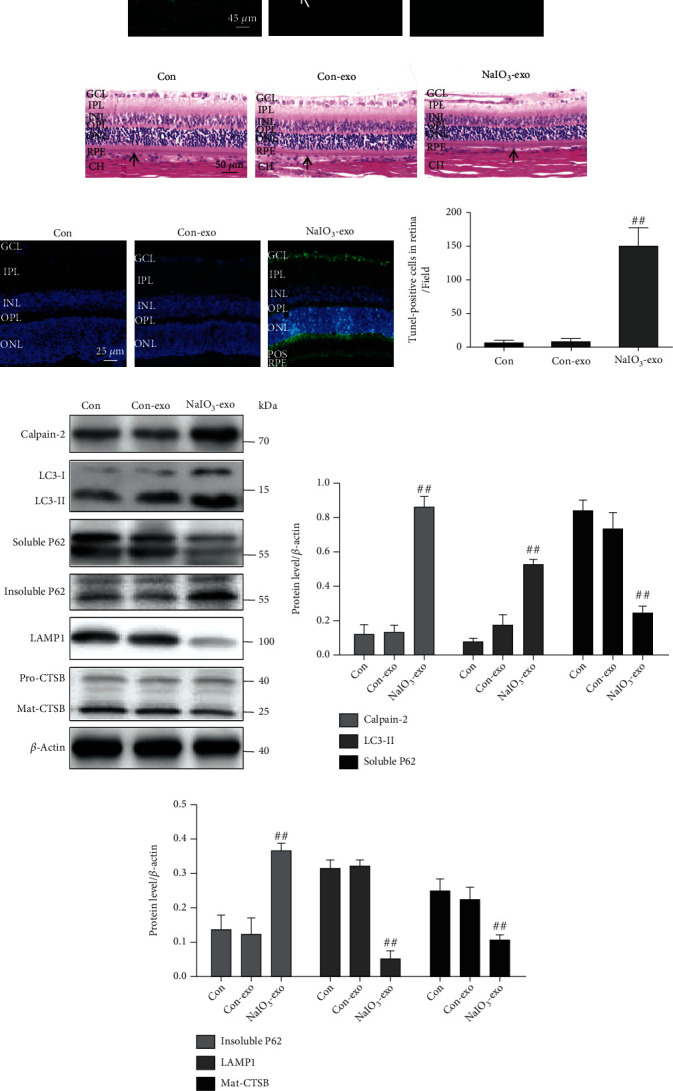
Exosome (NaIO_3_) induced calpain-2 activation, ALP dysfunction, and apoptosis in rat retina. (a) Exosome tracking analysis was used to assess the location of exosomes in the retina on day 0, day 7, and day 14 after injection. The white arrows are pointing toward the RPE cell layer. (b) HE staining and light micrographs were performed on the retinal cross-sections. The black arrows are pointing toward the RPE cells. (c, d) The TUNEL assay examined the retinal cell apoptosis in rats. GCL: ganglion cell layer; IPL: inner plexiform layer; INL: inner nuclear layer; OPL: outer plexiform layer; ONL: outer nuclear layer; POS: photoreceptor outer segments; RPE: retinal pigment epithelium; CH: choroid. (e–g) Calpain-2 and ALP-related proteins were examined by western blotting. ^#^*P* < 0.05, ^##^*P* < 0.01 vs. Con (control group).

To determine the dynamic distribution of NaIO_3_-exo in the retina, NaIO_3_-exos were labeled with DiO before injection. NaIO_3_-exos started to appear in the retina at 1 h after injection. They diffused throughout the retinal pigment epithelium (RPE) at 1 h and gradually spread to the retinal ganglion cells (RGCs). They showed no green fluorescence on day 14 (Figure 7(a)).

Next, we examined the effects of the NaIO_3_-exo on the retinal injury. On postinjection day 14, images of HE staining revealed that RGCs and RPEs showed low numbers and disordered nuclei in the NaIO_3_-exo group. Furthermore, there were a lot of RPEs that seemed to have moved from the subretinal space to the choroid (Figure 7(b)). The TUNEL assay showed that, compared with the control group, the cell apoptosis rate markedly increased in the NaIO_3_-exo group. A comparison between the control and con-exo group showed no significant differences (Figures 7(c) and 7(d)).

In addition, the expression levels of calpain-2 and ALP-related proteins were examined by western blotting in the retina. As shown in Figures 7(e)–7(g), compared with the control group, calpain-2 and ALP-related proteins significantly increased by NaIO_3_-exo. These data suggest that NaIO_3_-exo enhanced calpain-2 expression, ALP dysfunction, apoptosis, and retinal damage in rats. These results are consistent with the in vitro data.

## 4. Discussion

Sodium iodate (NaIO_3_) is widely used to simulate the damage during progressive AMD. In treatment with NaIO_3_, retinal pigment epithelium (RPE) is generally accepted as the primary cause of lesions, and photoreceptors are the second one contributing to visual impairment [23, 24]. In the previous study, we evaluated the toxic effect of NaIO_3_ on ARPE-19 cells at different doses. The results indicate that NaIO_3_ induced calpain activation, autophagy, and cell apoptosis in ARPE-19 cells. It should be noted in particular that exosomes derived from RPE cells under NaIO_3_ stimulation mediated autophagy, apoptosis, and other signaling pathways led to RPE injury in this study, rather than NaIO_3_. Because the exosome concentration selected in this study was 40 *μ*g·ml^−1^, 2.5 mM (800 *μ*g·ml^−1^) of sodium iodate, it did not affect ARPE-19 cells (Figure 2 in the Data Supplement).

ARPE-19 is a line of human RPE cells that show differentiating properties of RPE in vivo. To verify that ARPE-19 cells preserved their RPE phenotype, RPE65 immunofluorescence was performed. The expression of RPE65 confirmed that ARPE-19 preserved their RPE phenotype (Figure 3A in the Data Supplement). Staining of ZO-1 revealed an overall “cobblestone” appearance and intercellular tight junctions in ARPE-19 cells (Figure 3B in the Data Supplement). A TER assay was used to evaluate the integrity of the RPE barrier. The TER in DMEM-F12 with 1% FBS gradually increased and reached a plateau at 46.33 *Ω*·cm^−2^ after 4 weeks of culture, which was similar to the original TER report for this medium (Figure 3C in the Data Supplement) [25–31]. Our results demonstrate that ARPE-19 cells have structural and functional properties characteristic of RPE cells in vivo and suggest that this cell line could be used for in vitro studies of retinal pigment epithelium physiology.

Exosomes are released outside the cell by exocytotic activity. The released exosomes carry double-stranded DNA, mRNA, noncoding RNA (microRNA and lncRNA), and undigested or partially digested proteins out of the cell. This suggests that exosomes act as vital mediators during intercellular communication [32, 33]. Exosomes perform important roles in both physiological and pathological processes in AMD [34, 35]. Recent analysis has indicated that exosomes have therapeutic effects on pathological retinal angiogenesis [36]. However, some studies showed that, under certain conditions, exosomes mediate inflammation and apoptosis during retinal injury [5, 6]. In addition, it has been shown that in aged RPE in vivo, there is an age-related increased exocytotic activity leading to the release of intracellular proteins via exosomes, which contribute to the formation of drusen [37]. We hypothesize that local delivery of NaIO_3_-exo (exosomes from RPE cells under NaIO_3_ stimulation) could be harmful to normal ARPE-19 cells. The extracted exo or NaIO_3_-exo was cocultured with ARPE-19 cells for 24 h. The data showed that NaIO_3_-exo induced calpain activation, autophagy, and cell apoptosis in ARPE-19 cells, which is consistent with the toxic effect of NaIO_3_. For further evaluation of the exosome role in AMD, specific components of exosomes secreted by injured RPE cells should be targeted in future studies.

It is well known that abnormal autophagy and apoptosis contribute to the development of AMD [38, 39]. Our data showed that the dying cells displayed a large-scale accumulation of autophagosomes following NaIO_3_ insult. NaIO_3_-exo significantly promoted apoptosis in ARPE-19 cells. These results support the idea that RPE cell death is potentially related to autophagy.

An increasing number of researchers have investigated the autophagic flux and autophagosomes using various AMD models [40]. Here, we explored the dynamic change of the autophagy-lysosomal pathway (ALP) function induced by NaIO_3_-exo in both ARPE-19 cells and rats. Western blot analysis of cell lysates prepared in Triton X-100-containing lysis buffers under autophagic conditions shows a reduction in P62 levels. However, this does not necessarily indicate that P62 is degraded because P62 aggregates are insoluble under these particular detergent lysis conditions [41]. Therefore, both Triton X-100-insoluble and Triton X-100-soluble P62 fractions were investigated in our study. We found that NaIO_3_-exo markedly upregulated the expression of the autophagic marker LC3II and the substrate insoluble P62 but downregulated the soluble P62 level in ARPE-19 cells.

The accumulation of the LC3II protein or autophagosomes can be related to the decreased lysosomal degradation of autophagosomes [41, 42] because autophagosomes fuse with and are then degraded by lysosomes. Therefore, we investigated whether lysosomal dysfunction and defective autophagosome/lysosome fusion could be present. In our study, the protein expression of the lysosomal marker Mat-CTSB decreased from 6 h to 24 h. However, the LAMP1 expression significantly increased at 6 h and then gradually decreased compared with the control group, suggesting that lysosomal function was induced at the early stage, as reflected by the higher expression of the lysosomal marker LAMP1, which then declined at later stages. V-ATPase is a proton pump that establishes and maintains the acidic environment of lysosomes [21]. VAMP7 is a member of the SNARE complex that is responsible for the fusion of lysosomes with autophagosomes [22]. We could show that the expression of V-ATPase and VAMP7 significantly decreased compared with the control group. Thus, we suppose that NaIO_3_-exo increased the abnormal accumulation of autophagosomes and substrates by impairing lysosomal function and reducing autophagosome/lysosome fusion.

To further confirm this hypothesis, we treated ARPE-19 cells with CQ (an agent that prevents lysosomal acidification) or 3-MA. It must be pointed out that 3-MA may have dual roles in autophagy. It has been shown that 3-MA promotes autophagyic flux in nutrient-rich models, while others have shown that 3-MA inhibits autophagy during starvation [43]. Our data showed that no significant change was detected in the expression of calpain-2 and ALP-related proteins treated with CQ. However, ALP-related proteins could be reduced by 3-MA. Thus, these data confirmed that the increase in autophagic substrates was due to lysosomal dysfunction and did not affect the normal baseline autophagic pathway proteins.

Evidence has shown that calcium-dependent calpain protease activation can enhance apoptosis, which is mediated by multiple triggers, including aberrant endoplasmic reticulum stress, oxidative stress, metabolic alterations, and amyloid beta aggregation [44, 45]. Calpain activation induced permeabilization of the lysosomal membrane and consequent photoreceptor cell death in retinitis pigmentosa [41]. LAMP1 is crucial for the completion step of autophagy. Secreted hydrolases such as CTSB assist in the maturation of growth factors and the degradation of extracellular matrix components, facilitating cell proliferation and tumor invasion [46–48]. Calpain-mediated proteolysis of lysosomal proteins such as V-ATPase, LAMP1, and CTSB occurs in both pkd1−/- cells and primary epithelial cells [14]. Our finding that knocking down calpain-2 (si-C2) or the calpain-2 blocker calpain-2-IN-1 partially rescues lysosomal function and prevents RPE apoptosis indicates that calpain-2 plays a key role in RPE damage. To further evaluate the role of calpain-2 in AMD, studies of female and other AMD models and species should be considered in future research.

**Figure 8:**
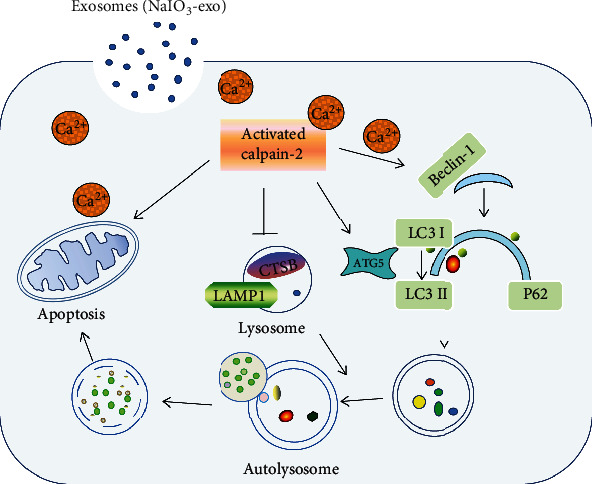
Schematic description of NaIO_3_-exo induced autophagic/lysosomal defects and apoptosis by activating calpain-2 in ARPE-19 cells and rats.

In conclusion, we discovered for the first time that NaIO_3_-exo induced calpain-2 activation, autophagy, lysosomal dysfunction, and cell apoptosis in ARPE-19 cells. The increase in autophagic substrates is related to lysosomal dysfunction. Furthermore, NaIO_3_-exo–activated calpain-2 participates in the ALP function and apoptosis in ARPE-19 cells. In addition, NaIO_3_-exo enhanced calpain-2 expression, ALP dysfunction, apoptosis, and retinal damage in rats. These results are in accordance with the in vitro data. Collectively, our present study provides a mechanistic link between calpain-2 and impaired ALP function and identifies calpain-2 as a promising molecular target for AMD therapy (Figure 8).

## Data Availability

All data included in this study are available upon request by contact with the corresponding author.

## Supplementary Materials

Supplementary Materials Supplemental Figure 1: the dynamic changes of calcium fluxes, calpain-2, autophagic flux, and lysosome function in con and con-exo groups at 0 h, 6 h, 12 h, and 24 h. Supplemental Figure 2: NaIO_3_ induced dose-specific calpain-2 activation, autophagy, and apoptosis in ARPE-19 cells. Supplemental Figure 3: immunofluorescence and TER of ARPE-19 cells.

## References

[1] Xing Y., Liang S., Zhao Y., Yang S., Ni H., Li H. (2021). Protection of Aronia melanocarpa fruit extract from sodium-iodate-induced damages in rat retina. *Nutrients*.

[2] Chen W., Ye Y., Wu Z. (2021). Temporary upregulation of Nrf2 by naringenin alleviates oxidative damage in the retina and ARPE-19 cells. *Oxidative Medicine and Cellular Longevity*.

[3] Shen Y., Zhuang P., Chiou G. C. (2011). Effect of guanabenz on rat AMD models and rabbit choroidal blood flow. *Open Ophthalmol J*.

[4] Wang Y., Zhang Q., Yang G., Wei Y., Li M., Du E. (2021). RPE-derived exosomes rescue the photoreceptors during retina degeneration: an intraocular approach to deliver exosomes into the subretinal space. *Drug Delivery*.

[5] Zhang W., Ma Y., Zhang Y., Yang J., He G., Chen S. (2019). Photo-oxidative blue-light stimulation in retinal pigment epithelium cells promotes exosome secretion and increases the activity of the NLRP3 inflammasome. *Current Eye Research*.

[6] Ke Y., Fan X., Rui H. (2020). Exosomes derived from RPE cells under oxidative stress mediate inflammation and apoptosis of normal RPE cells through Apaf1/caspase-9 axis. *Journal of Cellular Biochemistry*.

[7] Kiuchi K., Yoshizawa K., Shikata N., Moriguchi K., Tsubura A. (2002). Morphologic characteristics of retinal degeneration induced by sodium iodate in mice. *Current Eye Research*.

[8] Gupta U., Ghosh S., Wallace C. T. (2023). Increased LCN2 (lipocalin 2) in the RPE decreases autophagy and activates inflammasome-ferroptosis processes in a mouse model of dry AMD. *Autophagy*.

[9] Zhang Y., Ren S., Liu Y., Gao K., Liu Z., Zhang Z. (2017). Inhibition of starvation-triggered endoplasmic reticulum stress, autophagy, and apoptosis in ARPE-19 cells by taurine through modulating the expression of calpain-1 and calpain-2. *Int J Mol Sci*.

[10] Balmer J., Zulliger R., Roberti S., Enzmann V. (2015). Retinal cell death caused by sodium iodate involves multiple caspase-dependent and caspase-independent cell-death pathways. *Int J Mol Sci*.

[11] Wang W. Y., Xie L., Zou X. S. (2021). Inhibition of extracellular signal-regulated kinase/calpain-2 pathway reduces neuroinflammation and necroptosis after cerebral ischemia-reperfusion injury in a rat model of cardiac arrest. *International Immunopharmacology*.

[12] Wang Y., Liu Y., Yahya E., Quach D., Bi X., Baudry M. (2021). Calpain-2 activation in mouse hippocampus plays a critical role in seizure- induced neuropathology. *Neurobiology of Disease*.

[13] Zhang Y., Ren S., Gu Y., Wang J., Liu Z., Zhang Z. (2018). Taurine attenuates calpain-2 induction and a series of cell damage via suppression of NOX-derived ROS in ARPE-19 cells. *Oxidative Medicine and Cellular Longevity*.

[14] Peintner L., Venkatraman A., Waeldin A., Hofherr A., Busch T., Voronov A. (2021). Loss of PKD1/polycystin-1 impairs lysosomal activity in a CAPN (calpain)-dependent manner. *Autophagy*.

[15] Zhao Q., Guo Z., Deng W., Fu S., Zhang C., Chen M. (2016). Calpain 2-mediated autophagy defect increases susceptibility of fatty livers to ischemia-reperfusion injury. *Cell Death Dis*.

[16] Li Y., He Z., Lv H., Chen W., Chen J. (2020). Calpain-2 plays a pivotal role in the inhibitory effects of propofol against TNF-*α*-induced autophagy in mouse hippocampal neurons. *Journal of Cellular and Molecular Medicine*.

[17] Liu H., Dai L., Wang M., Feng F., Xiao Y. (2021). Tunicamycin induces hepatic stellate cell apoptosis through calpain-2/ca(2 +)-dependent endoplasmic reticulum stress pathway. *Frontiers in Cell and Development Biology*.

[18] Zhang H., Chang L., Zhang H. (2017). Calpain-2/p35-p25/Cdk5 pathway is involved in the neuronal apoptosis induced by polybrominated diphenyl ether-153. *Toxicology Letters*.

[19] Choi J. A., Kim Y. J., Seo B. R., Koh J. Y., Yoon Y. H. (2018). Potential role of zinc dyshomeostasis in matrix metalloproteinase-2 and -9 activation and photoreceptor cell death in experimental retinal detachment. *Investigative Ophthalmology & Visual Science*.

[20] Lynn S. A., Johnston D. A., Scott J. A., Munday R., Desai R. S., Keeling E. (2021). Oligomeric Abeta1-42 induces an AMD-like phenotype and accumulates in lysosomes to impair RPE function. *Cells*.

[21] Marshansky V., Futai M. (2008). The V-type H^+^-ATPase in vesicular trafficking: targeting, regulation and function. *Current Opinion in Cell Biology*.

[22] Fader C. M., Sanchez D. G., Mestre M. B., Colombo M. I. (2009). TI-VAMP/VAMP7 and VAMP3/cellubrevin: two v-SNARE proteins involved in specific steps of the autophagy/multivesicular body pathways. *Biochimica et Biophysica Acta*.

[23] Hanus J., Anderson C., Sarraf D., Ma J., Wang S. (2016). Retinal pigment epithelial cell necroptosis in response to sodium iodate. *Cell Death Discov*.

[24] Kannan R., Hinton D. R. (2014). Sodium iodate induced retinal degeneration: new insights from an old model. *Neural Regen Res*.

[25] Dunn K. C., Aotaki-Keen A. E., Putkey F. R., Hjelmeland L. M. (1996). ARPE-19, a human retinal pigment epithelial cell line with differentiated properties. *Experimental Eye Research*.

[26] Chen Y., Yang P., Li F., Kijlstra A. (2011). The effects of Th17 cytokines on the inflammatory mediator production and barrier function of ARPE-19 cells. *PLoS One*.

[27] Liu Y., Yamagishi R., Honjo M. (2022). Role of autotaxin in high glucose-induced human ARPE-19 cells. *Int J Mol Sci*.

[28] Zhang F., Liu L., Zhang H., Liu Z. L. (2020). Effect of platelet-activating factor on barrier function of ARPE-19 cells. *Drug Des Devel Ther*.

[29] Deissler H. L., Stutzer J. N., Lang G. K., Grisanti S., Lang G. E., Ranjbar M. (2020). VEGF receptor 2 inhibitor nintedanib completely reverts VEGF- A_165_-induced disturbances of barriers formed by retinal endothelial cells or long-term cultivated ARPE-19 cells. *Experimental Eye Research*.

[30] Alaimo A., Di Santo M. C., Dominguez Rubio A. P., Chaufan G., Garcia Linares G., Perez O. E. (2020). Toxic effects of A2E in human ARPE-19 cells were prevented by resveratrol: a potential nutritional bioactive for age-related macular degeneration treatment. *Archives of Toxicology*.

[31] Saenz-de-Viteri M., Fernández-Robredo P., Hernández M. (2016). Single- and repeated-dose toxicity study of bevacizumab, ranibizumab, and aflibercept in ARPE-19 cells under normal and oxidative stress conditions. *Biochemical Pharmacology*.

[32] Salmaninejad A., Pourali G., Shahini A., Darabi H., Azhdari S. (2022). MicroRNA and exosome in retinal-related diseases: their roles in the pathogenesis and diagnosis. *Combinatorial Chemistry & High Throughput Screening*.

[33] Liu J., Jiang F., Jiang Y. (2020). Roles of exosomes in ocular diseases. *International Journal of Nanomedicine*.

[34] Wassmer S. J., Carvalho L. S., Gyorgy B., Vandenberghe L. H., Maguire C. A. (2017). Exosome-associated AAV2 vector mediates robust gene delivery into the murine retina upon intravitreal injection. *Scientific Reports*.

[35] Yu B., Shao H., Su C. (2016). Exosomes derived from MSCs ameliorate retinal laser injury partially by inhibition of MCP-1. *Scientific Reports*.

[36] Dong X., Lei Y., Yu Z. (2021). Exosome-mediated delivery of an anti-angiogenic peptide inhibits pathological retinal angiogenesis. *Theranostics.*.

[37] Wang A. L., Lukas T. J., Yuan M., Du N., Tso M. O., Neufeld A. H. (2009). Autophagy and exosomes in the aged retinal pigment epithelium: possible relevance to drusen formation and age-related macular degeneration. *PLoS One*.

[38] Xie X., Li D., Cui Y., Xie T., Cai J., Yao Y. (2022). Decorin protects retinal pigment epithelium cells from oxidative stress and apoptosis via AMPK-mTOR-regulated autophagy. *Oxid Med Cell Longev*.

[39] Li S., Jiang Y., Xing X., Lin R., Li Q., Zhou W. (2021). Protective mechanism of berberine on human retinal pigment epithelial cells against apoptosis induced by hydrogen peroxide via the stimulation of autophagy. *Oxid Med Cell Longev*.

[40] Chu Y. K., Lee S. C., Byeon S. H. (2013). VEGF rescues cigarette smoking-induced human RPE cell death by increasing autophagic flux: implications of the role of autophagy in advanced age-related macular degeneration. *Investigative Ophthalmology & Visual Science*.

[41] Daniel J. (2021). Guidelines for the use and interpretation of assays for monitoring autophagy (4th edition)(1). *Autophagy*.

[42] Chen P. M., Gombart Z. J., Chen J. W. (2011). Chloroquine treatment of ARPE-19 cells leads to lysosome dilation and intracellular lipid accumulation: possible implications of lysosomal dysfunction in macular degeneration. *Cell Biosci*.

[43] Wu Y. T., Tan H. L., Shui G. (2010). Dual role of 3-methyladenine in modulation of autophagy via different temporal patterns of inhibition on class I and III phosphoinositide 3-kinase. *J Biol Chem*.

[44] Xie R. J., Hu X. X., Zheng L. (2020). Calpain-2 activity promotes aberrant endoplasmic reticulum stress-related apoptosis in hepatocytes. *World Journal of Gastroenterology*.

[45] Yildiz-Unal A., Korulu S., Karabay A. (2015). Neuroprotective strategies against calpain-mediated neurodegeneration. *Neuropsychiatric Disease and Treatment*.

[46] Peintner L., Borner C. (2017). Role of apoptosis in the development of autosomal dominant polycystic kidney disease (ADPKD). *Cell and Tissue Research*.

[47] Eskelinen E. L. (2006). Roles of LAMP-1 and LAMP-2 in lysosome biogenesis and autophagy. *Molecular Aspects of Medicine*.

[48] Mrschtik M., Ryan K. M. (2015). Lysosomal proteins in cell death and autophagy. *The FEBS Journal*.

