# Supplementary material for: Calpain-2 Facilitates Autophagic/Lysosomal Defects and Apoptosis in ARPE-19 Cells and Rats Induced by Exosomes from RPE Cells under NaIO3 Stimulation
Source: Oxid Med Cell Longev. 2023 Jan 17;2023:3310621. doi: 10.1155/2023/3310621 (PMC9873447; doi:10.1155/2023/3310621)
Supplement: Supplementary Materials — Supplemental Figure 1: the dynamic changes of calcium fluxes, calpain-2, autophagic flux, and lysosome function in con and con-exo groups at 0 h, 6 h, 12 h, and 24 h. Supplemental Figure 2: NaIO3 induced dose-specific calpain-2 activation, autophagy, and apoptosis in ARPE-19 cells. Supplemental Figure 3: immunofluorescence and TER of ARPE-19 cells. [file 3310621.f1.docx]

**Supplementary description**

**NaIO_3_ dose-dependently induced calpain-2 activation, autophagy and apoptosis in ARPE-19 cells.**

**Supplemental Methods**

**Cell viability assay**

The viability of the ARPE-19 cells was measured using a cell counting kit-8 (CCK-8) assay (Sunbao Biotech). ARPE-19 cells were sub-cultured in 96-well plates at a seeding density of 1×10^5^ cells/well for 24 h. ARPE-19 cells were exposed to NaIO_3_ (0.5, 2.5, 5, 10, 20 mM) for 24 h or 10 mM NaIO_3_ for 3, 6, 12, 24, 48 h. Next, 100 μL CCK-8 (1:10 diluted with medium) were added to each well and cells were incubated at 37 °C for 2 h. Subsequently, the absorbance was measured at 450 nm using Multiskan GO Microplate Spectrophotometer (Thermo).

**Immunofluorescence measurements of RPE65 and zona occludens protein 1 (ZO-1)**

ARPE-19 cells were cultured on cover slips at a density of 1×10^5^ cells/well for 24 and 48 h. At each time point, the RPE marker RPE65 (1:500, abclonal, A9841) was visualized by immunofluorescence following the same protocol as described in 2.4. Nuclei were stained with DAPI.

ARPE-19 cells were deposited on transwell flters at a density of 5×10^4^ cells/well, and were grown in DMEM/F12, 1% FBS, for 28 days. Immunofluorescence was performed using a ZO-1 anti-rabbit antibody (1:1000, abcam, ab221547) on the 7th and 28th days.

**Transepithelial electrical resistance (TER) measurement**

ARPE-19 cells were deposited onto transwell flters at a density of 5×10^4^ cells/well in DMEM/F12, 1% FBS. The TER in each well was measured after 3, 7, 14, 21, 28, and 35 days using a Millicell-ERS instrument (MilliporeSigma). Cells were kept at room temperature for 15 min before measurement. The final resistance (Ω·cm^-2^) was calculated by subtracting the background resistance from the measured barrier resistance and then dividing it by the effective surface area of the filter membrane.

**Supplemental Figures and Figure legends**

**
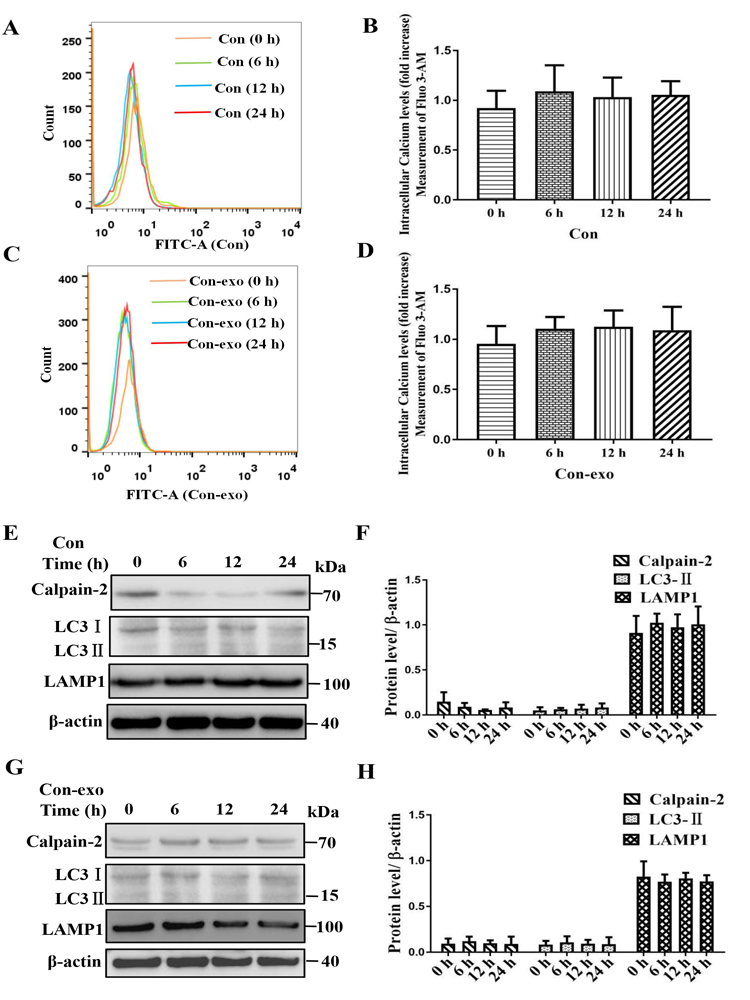
**

Supplemental Figure Ⅰ. The dynamic changes of calcium fluxes, calpain-2, autophagic flux, and lysosome function in con and con-exo groups at 0 h, 6 h, 12 h, and 24 h. (A-D) Flow cytometry was used to detect the dynamic changes of intracellular calcium ions in the con and con-exo groups at 0 h, 6 h, 12 h and 24 h. (E-H) Western blot analysis was performed to detect the expression levels of calpain-2, LC3, and LAMP1 in the con and con-exo groups at 0 h, 6 h, 12 h, and 24 h.


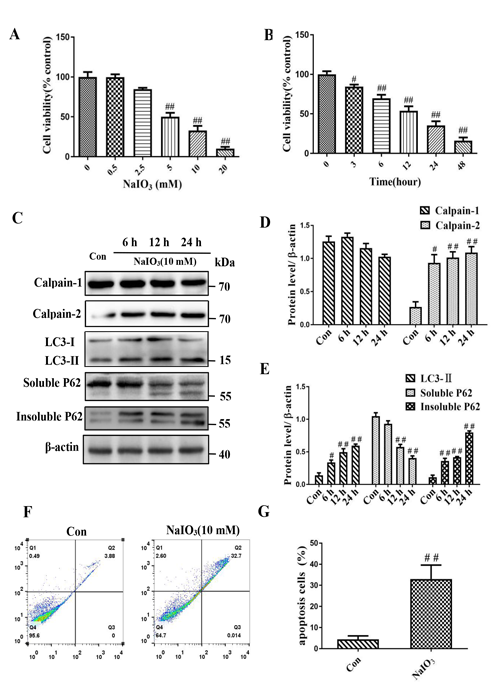


Supplemental Figure Ⅱ. NaIO_3_ induced dose specific calpain-2 activation, autophagy, and apoptosis in ARPE-19 cells. (A) NaIO_3_-induced cell apoptosis in a dose-dependent manner. (B) NaIO_3_-induced cell apoptosis in a time-dependent manner. (C) Western blot analysis was performed to detect the expression levels of calpain-1, calpain-2, LC3 and P62 in ARPE-19 cells. (D, E) Quantitative analysis of the immunoblotted proteins was performed with Image J. (F, G) The percentage of apoptotic cells was determined based on flow cytometric analysis. ^#^*P*＜0.05, ^##^*P*＜0.01 *vs* control.


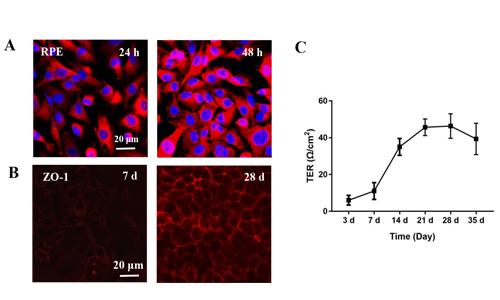


Supplemental Figure Ⅲ. Immunofluorescence and TER of ARPE-19 cells. A. Confocal microscope images of ARPE-19 cells labeled with anti-RPE65 at 24 h and 48 h. B. Confocal microscope images of ARPE-19 cells labeled with anti- ZO-1 at 7 day and 28 day. C. The transepithelial electrical resistance (TER) of a cultured ARPE-19 monolayer.
